# Editorial: Emerging frontiers in developmental biology in Latin America

**DOI:** 10.3389/fnins.2023.1129291

**Published:** 2023-04-20

**Authors:** Daniel Ortuño-Sahagún, Juan Rafael Riesgo-Escovar

**Affiliations:** ^1^Laboratorio de Neuroinmunobiología Molecular, Instituto de Investigación en Ciencias Biomédicas, Centro Universitario de Ciencias de la Salud, Universidad de Guadalajara, Jalisco, Mexico; ^2^Developmental Neurobiology and Neurophysiology, Instituto de Neurobiología, Campus UNAM Juriquilla, Universidad Nacional Autónoma de Mexico, Santiago de Querétaro, Mexico

**Keywords:** developmental biology, Latin America, neuroscience, model organisms, model system

These past couple of years have been a trying experience for us all; and we are dealing still with the aftermath of the pandemic and the lingering sequelae. That has caused numerous academic activities to be postponed, and the Latin American Society of Developmental Biology, as well as the Mexican Society of Developmental Biology meetings were not immune. Nevertheless, we have gone ahead and organized this Research Topic, and have had an enthusiastic response, in many ways a testament to the vigor and resilience in these difficult times of our members and research groups, and the excitement that permeates developmental biology in Latin America. Eleven papers constitute this Research Topic, ranging from single molecules to whole organisms, exploiting classical developmental biology models such as flies (*Drosophila melanogaster*), bees (*Apis mellifera*), and axolotls (*Ambystoma mexicanus*), to recent additions like echinoderms (*Holothuria glaberrima*), four-eyed fish (*Anableps anableps*), and organoids, enriching the developmental biology models' cornucopia, in what will hopefully be a first installment for envisioned future submissions and discoveries. We have no qualms that you, our readers, will find them interesting and motivating.

Most of the papers of the Research Topic deal with aspects of the developing nervous system. The central nervous system (CNS) has always generated great interest, an interest that is both historical and current (Pellet et al., 2022), going through topics such as embryonic development (Elshazzly et al., 2022), aging (Wolkow et al., 2010), and its regenerative capacity after injury (Buga et al., 2011).

Bolatto et al. examine *Patched-related (Ptr)* null mutant embryos in Drosophila. As a neuroectodermal gene, Ptr encodes for a protein with a Patched (Ptc)-like organization and domain structure, which is a canonical receptor of the Hedgehog (Hh) signaling pathway, and demonstrate that Ptr is required for proper CNS development. CRISPR/Cas editing has been widely employed in recent years for the understanding of CNS function (Cota-Coronado et al., 2019; Sandoval et al., 2020). Barragán-Álvarez et al., focusing on emerging methods, such as GESTALT and LINNAEUS, review recent advances in the application of the CRISPR/Cas system for the study of glial cell-associated neurological disorders and their potential applications in regenerative medicine.

CNS cells communicate mainly through specialized contact sites called synapses that use neurotransmitters to modulate neuronal activity (Andreae and Burrone, 2014) and circuit development (Andreae and Burrone, 2018). Among these neurotransmitters, Salceda reviews the functions of glycine and its receptors, present from early CNS development to adulthood, focusing on glycinergic activity in the balance of excitatory and inhibitory signals during development, cell proliferation, and specification.

With the neurosciences, neurogenesis is a very attractive topic. It occurs during both embryonic and postnatal development (reviewed in Bartkowska et al., 2022), where Notch signaling plays an important role (reviewed in Engler et al., 2018). Arzate et al. document the involvement of the delta-like 1 (Dll1) gene as a transmembrane ligand that activates the Notch receptor in the developing brain. By analyzing Dll1 haploinsufficiency in adult mice, they found brain abnormalities derived from reductions in neurogenesis, that may cause slight brain disfunction and mild behavioral alterations. In addition to genetics, studies of epigenetic mechanisms regulating neural plasticity have increased recently (Ortuño-Sahagún et al., 2019). Bataglia et al. describe how dietary changes influence CNS functioning by presenting tissue-specific signatures of RNA methyltransferases expression in adult honeybee workers.

Sensory stimulation can modulate CNS function, one of which is the visual system (Kovács-Öller et al., 2022). The four-eyed fish *Anableps anableps* possesses duplicate corneas and pupils, as well as specialized retinal regions associated with aerial or aquatic vision (Perez et al., 2017). Salgado et al. analyze the expression profile of several opsin genes in dorsal and ventral retinas. Asymmetry is established after birth and different light conditions can shift opsin expression, potentially contributing to changes in spectral sensitivity in this four eyed fish.

In addition to neurons, glial cells are fundamental for CNS proper functioning (Nampoothiri et al., 2022), as well as in neural pathologies (Mata-Martínez et al., 2022). Miranda-Negrón and García-Arrarás review how radial glia and radial glia-like cells participate in neurogenesis and regeneration during homeostasis and in response to injury, using the echinoderm *Holothuria glaberrima*.

Finally, Palma's review focuses on understanding the onset of schizophrenia from a neuro-angiogenic perspective. She emphasizes the role of the cerebral vasculature, during angiogenesis and during the establishment and functioning of the neurovascular niche, in relation to the onset of schizophrenia, considered a chronic mental disorder. It offers a perspective on distinctive angiogenic and neurogenic signaling pathways that might be involved in schizophrenia.

Moving outside the CNS, other areas of developmental biology are covered in this Research Topic: body patterning, limb regeneration, and *in vitro* organoid models. Through a detailed review, Mundaca-Escobar et al. discuss how Wnt signaling could regulate segmentation to establish a repetitive pattern, suggesting that this pathway plays an organizing role in the formation of body segments, regulating the expression of segmentation genes in most arthropods. Carbonell-M et al. show how reactive oxygen species generated after amputation are necessary for limb regeneration in the axolotl, showing that ROS/H_2_O_2_ are necessary for correct morphogenesis and size of skeletal structures, as well as proper integration between regenerated structures and remaining tissues. Finally, technical advances lead to the development of specialized *in vitro* culture techniques and organoid formation. The recombinant limb assay, developed by Zwilling (1964), is reviewed by García-García et al., proposing that the formation of skeletal elements induced through this system, when occurring from precursor cells, may resemble *in vivo* morphogenic properties of skeletal cells.

Overall, this Research Topic constitutes a sample of the areas of developmental biology cultivated in the region, and may serve as a kernel to detonate future collaborations and interactions, and to foster the rekindling of developmental biology studies throughout the region and elsewhere, especially at this trying post-pandemic times. It may also, hopefully, play its part in exciting and motivating a new generation of developmental biology students and scientists.

## Author contributions

DO-S and JR-E wrote the manuscript, designed the Research Topic, and reviewed the manuscript. Both authors contributed to the article and approved the submitted version.
